# ICU admission for solid cancer patients treated with immune checkpoint inhibitors

**DOI:** 10.1186/s13613-023-01122-z

**Published:** 2023-04-18

**Authors:** Anne-Claire Toffart, Anne-Pascale Meert, Florent Wallet, Aude Gibelin, Olivier Guisset, Frédéric Gonzalez, Amélie Seguin, Achille Kouatchet, Myriam Delaunay, Didier Debieuvre, Boris Duchemann, Gaëlle Rousseau-Bussac, Martine Nyunga, David Grimaldi, Albrice Levrat, Elie Azoulay, Virginie Lemiale

**Affiliations:** 1 Department of Pneumology and Physiology, CHU Grenoble Alpes, Grenoble, France; 2 Department of Internal Medicine, Institut Jules Bordet, Université Libre de Bruxelles (ULB), Brussels, Belgium; 3 Department of Resuscitation, Hospices Civils de Lyon, Lyon, France; 4 Department of Resuscitation, Hôpital Tenon, AP-HP, Paris, France; 5 Department of Intensive Care and Resuscitation, Hôpital Saint André, CHU Bordeaux, Bordeaux, France; 6 Resuscitation Unit, Institut Paoli-Calmettes, Marseille, France; 7 Department of Resuscitation, CHU de Nantes, Nantes, France; 8 Department of Resuscitation, CHU d’Angers, Angers, France; 9 Department of Pneumology, CHU de Toulouse, Toulouse, France; 10 Department of Pneumology, CH de Mulhouse, Mulhouse, France; 11 Department of Thoracic Oncology, Hôpital Avicenne, Bobigny, France; 12 Department of Pneumology, CH Intercommunal de Créteil, Créteil, France; 13 Department of Resuscitation, CH de Roubaix, Roubaix, France; 14 Department of Resuscitation, Cliniques Universitaires de Bruxelles - Hôpital Erasme, Université Libre de Bruxelles, Brussels, Belgium; 15 Department of Resuscitation, CH Annecy Genevois, Annecy, France; 16 Department of Resuscitation, Hôpital Saint-Louis, AP-HP, Paris, France

**Keywords:** Solid tumor, Immune checkpoint inhibitor, Intensive care, Immune-related adverse event

## Abstract

**Background:**

Immune checkpoint inhibitors (ICI) have revolutionized the management of cancer. They can induce immune-related adverse events (irAE) leading to intensive care unit (ICU) admission. We aimed to describe irAEs for ICU admissions in solid cancer patients treated with ICIs.

**Methods:**

This prospective multicenter study was conducted in France and Belgium. Adult patients with solid tumor and treated with systemic ICIs within the last 6 months, requiring non-programmed ICU admission were included. Patients admitted for microbiologically documented sepsis were excluded. Imputability of irAEs in ICU admissions was described according to the WHO-UMC classification system at ICU admission and at ICU discharge. The use of immunosuppressant treatment was reported.

**Results:**

115 patients were eligible. Solid tumor was mainly lung cancer (n = 76, 66%) and melanoma (n = 18, 16%). They were mainly treated with an anti-PD-(L)1 alone (n = 110, 96%). Main ICU admission reasons were acute respiratory failure (n = 66, 57%), colitis (n = 14, 13%), and cardiovascular disease (n = 13, 11%). ICU admission was considered “likely” associated with irAE for 48% (n = 55) of patients. Factors independently associated with irAE were a good ECOG performance status (PS) (ECOG-PS of 0 or 1 vs. ECOG-PS of 2–3, odds ratio [OR] = 6.34, 95% confidence interval [95% CI] 2.13–18.90, and OR = 3.66, 95% CI 1.33–10.03, respectively), and a history of irAE (OR = 3.28, 95% CI 1.19–9.01). Steroids were prescribed for 41/55 (75%) patients with ICU admission “likely” related to irAE. Three patients were subsequently treated with immunosuppressants.

**Conclusion:**

IrAEs accounted for half of ICU admissions in cancer patients receiving ICIs. They could be treated with steroids. Identifying the imputability of irAEs in ICU admissions remains a challenge.

**Supplementary Information:**

The online version contains supplementary material available at 10.1186/s13613-023-01122-z.

## Background

In the last decade, immune checkpoint inhibitors (ICI) have greatly improved cancer treatment and outcome for patients with cancer such as melanoma, lung cancer, kidney cancer, or head-and-neck cancer.

Checkpoint proteins (programmed death-ligand 1 [PD-L1] on tumor cells, programmed cell death 1 [PD-1] or cytotoxic T-lymphocyte antigen-4 [CTLA-4] on T cells) help to keep immune responses in check. Blocking these checkpoints by ICIs induces the immune response: the activation of antitumor T cells in a lymph node and their migration to the tumor tissue can lead to an objective tumor response [1]. The adverse events related to this therapeutic approach are called immune-related adverse events (irAE). They can affect multiple organs in the body (skin, gastro-intestinal tract, lung, endocrine, or other systems) [2]. Some of these side effects, such as myocarditis, colitis, or interstitial lung disease, can be severe (Common Terminology Criteria for Adverse Events [CTCAE] grade 3 or more) and lead to hospitalization in the intensive care unit (ICU). Early recognition of those effects seems essential in order to set up appropriate treatment.

Few studies described such adverse events. One recent published review described the management of such patients in the ICU [3]. A recent retrospective study conducted in cancer patients treated with ICIs and admitted to the ICU [4] reported that 26% of ICU admissions were related to irAEs, which were mainly pneumonitis. Compared to other reasons (intercurrent events or complications related to tumor progression), admissions for irAEs were associated with better outcome. Some case series were also published: on neurotoxicity [5] or on the management of steroid-refractory pneumonitis [6].

This prospective study aimed to describe irAEs for ICU admissions in solid cancer patients treated with ICIs.

## Methods

### Design and setting

This was a prospective, observational, multicenter study. Forty-one centers were asked for participation in France and Belgium. Study protocol has been approved by the Ethics Committee of the Jules Bordet Institute, Belgium (08/29/2018). According to French Law, there is an institutional review board waiver for this kind of research. Nonetheless, all patients had to sign an informed consent. Clinical trial ID is NCT03357861.

Our primary objective was to identify imputability of irAEs for critically ill cancer patients treated with ICIs. The secondary objectives were to report the management of such adverse events, the ICU, and hospital mortality rates.

### Study population

Between 08/29/2018 and 03/01/2020, consecutive patients aged ≥ 18 years with a diagnosis of solid tumor and treated with ICIs within the last 6 months, requiring non-programmed ICU admission at the participating centers were evaluated. We did not include patients with ICU stays < 24 h or unwillingness to participate in the study. Patients admitted for microbiologically documented sepsis (defined by infection, host response, and organ dysfunction) were also excluded since their ICU admission could not be obviously related to irAE.

ICIs are defined as drugs that target T-cell suppressive pathways and could include, but were not limited to: pembrolizumab and nivolumab (anti-PD-1), atezolizumab, durvalumab and avelumab (anti-PD-L1), ipilimumab and tremelimumab (anti-CTLA-4) given as single agents or in combination.

### Data collection

Variables related to characteristics of patients, cancer, and its treatment were recorded: sex, age, comorbidities, Eastern Cooperative Oncology Group* (*ECOG) performance status (PS) [13] during the month before ICU admission, cancer organ and metastatic status, ICI, previous immune-related toxicity (CTCAE grade ≥ 2), previous antitumor treatment, disease status (controlled [i.e. complete or partial response and stable disease], non-controlled, unknown).

Data related to ICU stay were year of admission, organ failures, Simplified Acute Physiology Score II (SAPS II), organ support during ICU stay, end-of-life decisions, symptoms potentially related to immunotherapy (type, length of symptoms before ICU admission, diagnosis strategy and management [antibiotics, immunosuppressive, or steroid treatment]). Admission reasons were divided into six categories: respiratory, colitis, cardiovascular, metabolic, neurological, and sepsis (except respiratory infection and colitis).

IrAE imputability was assessed at admission and discharge from the ICU with both the intensivist and the oncologist. Definition of irAEs was in accordance to the classification of World Health Organization-Uppsala Monitoring Centre (WHO-UMC) system (Additional file 1: Table S1) for standardized case causality assessment. Reviewers from the study group (ACT, VL, APM) independently proposed a categorization based on the data collected in the study. Discordant cases were reviewed together.

Outcome was recorded for ICU and hospital discharge, vital status at last follow-up.

### Statistical analyses

Data were expressed as number (and percentage) for qualitative variables and median (interquartile range [IQR] 25%–75%) for quantitative variables. Two groups were compared (event likely and unlikely related to irAE) using chi-squared or Wilcoxon tests, as appropriate.

As documented, sepsis was never considered as related to irAE, such sepsis at ICU admission were excluded from the analyses. Univariate and multivariate logistic regression analyses were performed to identify characteristics at ICU admission associated with irAE imputability assessed at ICU admission. Multivariate logistic regression was performed with all variables clinically relevant, and after a backward selection of variables yielding *P* values < 0.25 in univariate analysis. Missing data were indicated in the population description. They were imputed elsewhere by the variable median value (for quantitative variables) or the most common level (for qualitative variables) in the logistic regression. Survival curves were obtained using the Kaplan–Meier method and compared by means of the log-rank testing.

All tests were two-sided, and *P-*values < 0.05 were considered statistically significant. All statistical analyses were performed using SAS 9.4 (SAS Institute, Cary, NC, USA).

## Results

### Description of the cohort

**Fig. 1:**
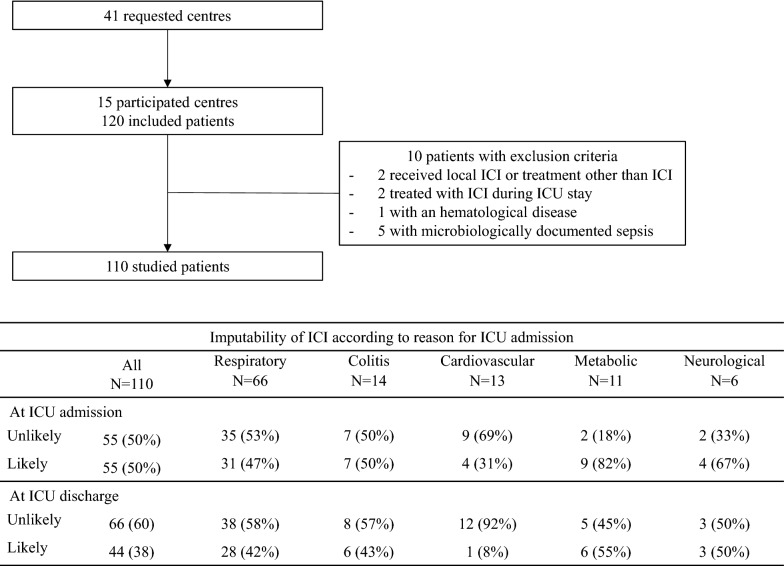
Study flowchart. *ICI* immune checkpoint inhibitor, *ICU* intensive care unit, *irAE* immune-related adverse event

From 15 centers, 120 patients were included (Fig. 1). Ten patients were excluded: five patients had microbiologically documented sepsis, two patients received intra-tumor ICIs or treatment other than ICIs (OSE2101 vaccine), one patient received ICIs for a hematological malignancy, and for two patients ICI was initiated during ICU stay. Finally, data of 110 patients were studied.

Patient characteristics according to reason for ICU admission are described in Table 1. Solid tumor was mainly lung cancer (n = 74, 67%) and melanoma (n = 18, 16%). Almost all patients had metastatic disease (n = 89, 81%). They were mainly treated with an anti-PD-(L)1 alone (n = 105, 95%), as 1st or 2nd line treatment (n = 94, 85%). A history of IrAE ≥ 2 prior to ICU admission was identified in 24 (22%) patients.

**Table 1:** Characteristics of patients according to proposed IrAE causality at ICU discharge (n = 110 patients)

	All n = 110	Unlikely causality n = 66 (60)	Likely causality n = 44 (38)	*P*-value
Male sex	73 (66)	44 (67)	29 (66)	0.93
Age (years)				
ECOG-PS (Miss. = 1)				0.001
0	29 (27)	12 (18)	17 (39)	
1	38 (35)	20 (31)	18 (41)	
2	29 (27)	22 (34)	8 (16)	
3	13 (12)	11 (17)	2 (5)	
CCI				
Chronic pulmonary disease	32 (29)	20 (30)	12 (27)	0.73
Type of cancer				0.59
Lung	74 (67)	46 (70)	28 (64)	
Melanoma	18 (16)	8 (12)	10 (23)	
Bladder	5 (5)	4 (6)	1 (2)	
Kidney	3 (3)	2 (3)	1 (2)	
Other	10 (9)	6 (9)	4 (9)	
Metastatic disease	89 (81)	54 (82)	35 (80)	0.77
Brain metastasis	80 (18)	13 (20)	7 (16)	0.61
Cancer status				0.22
Controlled	40 (36)	20 (30)	20 (45)	
In progression	33 (30)	23 (35)	10 (23)	
Not evaluated	37 (34)	23 (35)	14 (32)	
ICI characteristics				
Line of treatment				0.37
1 or 2	94 (85)	58 (88)	36 (82)	
> 2	16 (15)	8 (12)	8 (18)	
ICI				0.16
Anti-PD-1/-L1 alone	105 (95)	65 (98)	40 (91)	
Combination with anti-CTLA4	5 (5)	1 (2)	4 (9)	
History of irAE ≥ 2 before ICU admission	24 (22)	10 (15)	14 (32)	0.04
Time from first ICI infusion (months)	71 (28–165)	69 (19–144)	78 (34–185)	0.31

Qualitative variables are expressed as n (%) and quantitative variables as median [interquartile range 25–75%]

*CCI* Charlson Comorbidity Index, *CTLA4* cytotoxic T-lymphocyte antigen-4, *ECOG* Eastern Cooperative Oncology Group, *ICI* immune checkpoint inhibitor, *ICU* intensive care unit, *irAE* immune-related adverse event, *Miss*. missing data, *PD-1* programmed cell death 1, *PD-L1* programmed death-ligand 1, *PS* performance status

### Reasons for ICU admission

ICU admission reasons were acute respiratory failure (n = 66, 60%), colitis (n = 14, 13%), cardiovascular disease (n = 13, 12%), metabolic disorder (n = 11, 10%), and neurological disease (n = 6, 6%) (Additional file 1: Table S2).

Acute respiratory failure was mostly interstitial pneumonia (n = 52) (Table S2). It was secondary to myositis in two patients and to macrophagic activation syndrome in another. Two patients had pneumonitis associated with myocarditis. For the other patients, acute respiratory failure was directly due to acute chest disease. For no other patient was more than one severe irAE reported.

Patients with cardiovascular diseases had cardiac tamponade (n = 5), pulmonary embolism (n = 2), heart rhythm or conduction disorder (n = 2), acute coronary syndrome (n = 1), and arterial hypertension (n = 1).

### Imputability of irAEs in ICU admissions (Fig. 1)

The investigators performed a binary classification of imputability for irAE, that allowed full agreement. Causality categories were pooled into two categories: likely for “certain”, “probable/likely”, and “possible” and unlikely for “unlikely”, “conditional/unclassified”, and “unassessable/unclassifiable”.

As explained in the Methods section, the five patients admitted for sepsis were excluded of the analyses because none of their ICU admission was related to irAE.

At ICU admission, events were “likely” related to irAE for 31 (47%) of the 66 patients admitted with acute respiratory failure (Fig. 1), and for respectively 81%, 67%, 50% and 31% of patients admitted to ICU with metabolic disorder, neurological failure, colitis and cardiovascular disease.

At ICU discharge, 12 patients with ICU admission initially considered as “likely” associated with ICI were reclassified as “unlikely” (Fig. 1). One patient with pneumonia, one with encephalitis, and two with colitis had finally infectious disease. For three patients with pericardial effusion, two with pneumopathy and one with renal failure, cancer progression was finally (at ICU discharge) deemed the reason for ICU admission. For two patients, acute kidney injury was finally not considered as associated with immune toxicity.

One patient initially classified as “unlikely” was reclassified. He was admitted for febrile diarrhea, finally considered as colitis regarding history of hypophysitis with ICI.

Twenty-four patients had a history of immune toxicity before ICU admission. Among the 55 patients with an ICU admission initially considered as “likely” associated with ICI, a history of immune toxicity was reported for 14 (25%) of them. At ICU discharge, 14 of the 44 (32%) patients with an ICU admission finally considered as “likely” associated with ICI had a history of immune toxicity.

### ICU management of the patients

Severity of the patients was similar in both groups (Table 2). There was no difference s observed regarding the use of organ support during ICU stay.

**Table 2:** Characteristics of ICU stay according to proposed IrAE causality at ICU discharge (n = 110 patients)

	All n = 110	Unlikely causality n = 66 (60)	Likely causality n = 44 (38)	*P*-value
At ICU admission				
SAPS II	40 [34–50] Miss. = 10	42 [34–49] Miss. = 5	39 [34–52] Miss. = 5	0.84
Reason for ICU admission				0.09
Respiratory	66 (60)	38 (58)	28 (64)	
Colitis	14 (13)	8 (12)	6 (14)	
Cardiovascular	13 (12)	12 (18)	1 (2)	
Metabolic	11 (10)	5 (8)	6 (14)	
Neurologic	6 (5)	3 (5)	3 (7)	
During ICU stay				
Corticosteroids	54 (49)	17 (26)	37 (84)	< 10^–4^
Vasopressor	26 (24)	14 (21)	12 (27)	0.46
High flow oxygen	41 (37)	20 (30)	21 (48)	0.06
NIV	22 (24)	14 (21)	8 (18)	0.70
IV	25 (23)	13 (20)	12 (27)	0.35
ECMO	1 (1)	1 (2)	0	…
Dialysis	6 (5)	3 (5)	3 (7)	…
Treatment-limitation decisions	54 (49)	35 (53)	19 (43)	0.31

Qualitative variables are expressed as n (%) and quantitative variables as median [interquartile range 25–75%]

*ECMO*, extracorporal membrane oxygenation; *ICU* intensive care unit; *irAE* immune-related adverse event; *IV* invasive ventilation; *NIV* non-invasive mechanical ventilation; *Miss.*, missing data; SAPS, simplified acute physiology score

Steroids were largely used for the patients with ICU admission “likely” related to irAE (41/55, 75%). The four patients with a certain causality at ICU admission received steroids, as well as 18 of the 20 patients with probable causality. The 2/20 patients with probable causality who were not treated with steroids had a final diagnosis of hemoptysis and pneumocystis.

Steroids were initiated before ICU admission for three patients (three patients with missing data). Among the 35 patients who started steroids during ICU stay, 20 patients received steroids the day of ICU admission. Length from ICU admission to steroids was 0 day [IQR 25–75% 0–2] (missing data, n = 3). Three patients were subsequently treated with other immunosuppressants. One had a thrombotic microangiopathy (treated by rituximab). He died after 3 days in ICU. Another patient had a pneumonitis associated with a myocarditis (treated by abatacept). He died 3 days after ICU admission. The last patient had a pneumonitis complicated with a myocarditis (treated by infliximab). He was discharged alive 24 days after ICU admission and died within 6 weeks during hospitalization.

### Factors associated with imputability of irAEs in ICU admissions

After selection of variables in multivariate analysis, a good ECOG-PS (ECOG-PS of 0 or 1 vs. ECOG-PS of 2–3, OR = 6.34, 95% CI 2.13–18.90, and OR = 3.66, 95% CI 1.33–10.03, respectively) and a history of irAE (OR = 3.28, 95% CI 1.19–9.01) were independently associated with ICU admission “likely” related with irAE.

In multivariate analysis with variables clinically relevant, in addition to ECOG-PS, an admission for cardiovascular failure was less associated with an ICU admission “likely” related with irAE compared to respiratory failure (OR = 0.03, 95% CI 0.003–0.043) (Table 3).

**Table 3:** Factors associated with irAE at ICU discharge in patients receiving ICI and admitted to the ICU (n = 110 patients)

	Univariate analysis		Multivariate analysis with variables clinically relevant		Multivariate analysis with backward selection	
	Odds ratio (95% CI)	*P*-value	Odds ratio (95% CI)	*P*-value	Odds ratio (95% CI)	*P*-value
Gender: female vs. male	1.04 (0.46–2.32)	0.93	0.93 (0.34–2.53)	0.89		
Age (per year)	1.02 (0.98–1.06)	0.33	1.01 (0.99–1.10)	0.15		
CCI (per point)	0.91 (0.79–1.06)	0.23	0.94 (0.78–1.14)	0.54		
ECOG-PS		0.005		0.0005		0.003
2–3	1		1		1	
1	3.40 (1.27–8.99)		8.43 (2.39–29.72)		3.66 (1.33–10.03)	
0	5.35 (1.89–15.17)		11.23 (2.87–43.97)		6.34 (2.13–18.90)	
Type of cancer		0.61		0.63		
Lung	1		1			
Melanoma	2.05 (0.73–5.82)		0.67 (0.55–12.90)			
Bladder	0.41 (0.04–3.86)		0.31 (0.01–7.14)			
Kidney	0.82 (0.07–9.48)		2.47 (0.16–37.43)			
Other	1.10 (0.28–4.22)		1.08 (0.187–6.17)			
Metastatic vs. localized disease	0.86 (0.33–2.26)	0.77		0.65		
Cancer status		0.22		0.14		
Controlled	1		1			
In progression	0.44 (0.17–1.14)		0.30 (0.08–1.15)			
Not evaluated	0.61 (0.25–1.51)		1.22 (0.37–4.04)			
Reason for ICU admission		0.27		0.12		
Respiratory	1		1			
Colitis	1.02 (0.32–3.27)		0.57 ( 0.12–2.74)			
Cardiovascular	0.11 (0.01–0.92)		0.03 (0.003–0.043)			
Metabolic	1.63 (0.45–5.88)		1.00 (0.17–6.02)			
Neurologic	1.36 (0.26–7.23)		0.35 (0.03–3.69)			
Line of anticancer treatment: > 2 vs. 1–2	1.61 (0.56–4.67)	0.38	2.06 (0.46–9.20)	0.35		
History of immune toxicity: yes vs. no	2.61 (1.04–6.59)	0.04	4.13 (0.88–19.50)	0.07	3.28 (1.19–9.01)	0.02
Time from first ICI infusion (per month)	1.02 (0.97–1.07)	0.43	0.94 (0.87–1.01)	0.08		

*CCI* Charlson Comorbidity Index, *CI* confidence interval, *ECOG* Eastern Cooperative Oncology Group, *ICI* immune checkpoint inhibitor, *ICU* intensive care unit, *PS* performance status

### Outcome

Overall ICU mortality was 21% (n = 23/110), 22% (n = 12/55) for the patients with ICU admission “likely” related to irAE, and 20% (n = 11/55) for the others. It was 28% (19/66) for patients with respiratory diseases and 27% (3/11) for those with metabolic disorders (Table 4). No death was observed for patients admitted with colitis or cardiovascular disease.

**Table 4:** Length of ICU and hospital stay according to reason for ICU admission

	Respiratory N = 66	Colitis N = 14	Cardiovascular N = 13	Metabolic N = 11	Neurological N = 6
Time to ICU discharge (days)	5 [3–11]	4 [3–10]	13 [8–17]	3 [2–5]	5 [3–12]
Admission “likely” related to irAE	7 [4–14]	3 [3–5]	4 [3–7]	3 [3–5]	8 [4–38]
Admission “unlikely” related to irAE	4 [2–8]	4 [2–12]	2 [1–4]	2 [2–2]	4 [2–6]
ICU mortality	19 (28)	0 (0)	0 (0)	3 (27)	1 (17)
Admission “likely” related to irAE	9 (47)			2 (67)	1 (100)
Admission “unlikely” related to irAE	10 (53)			1 (33)	0 (0)
Time to hospital discharge (days)	14 [7–19]	14 [10–43]	13 [8–17]	8 [3–14]	13 [12–20]
Admission “likely” related to irAE	14 [9–21]	22 [9–48]	13 [5–25]	8 [5–8]	13 [12–55]
Admission “unlikely” related to irAE	9 [5–17]	12 [10–19]	13 [9–15]	10 [2–12]	16 [12–20]
Hospital mortality	29 (44)	2 (14)	3 (23)	3 (27)	1 (17)
Admission “likely” related to irAE	12 (41)	0 (0)	0 (0)	2 (67)	1 (100)
Admission “unlikely” related to irAE	17 (59)	2 (100)	3 (100)	1 (33)	0 (0)

Qualitative variables are expressed as n (%) and quantitative variables as median [interquartile range 25%–75%]

^*^In nine patients steroids were started before ICU admission

*ICU* intensive care unit, *irAE* immune-related adverse event

Hospital mortality was 27% (15/55) for the patients with ICU admission “likely” related to irAE, and 42% (n = 23/55) for the others. It was 44% (29/66) for patients with lung adverse event.

## Discussion

In this study, half of the patients treated with ICI for solid cancer and admitted to ICU had an irAE. All patients with a certain or probable irAE at ICU discharge received steroids. Only three patients were treated with other immunosuppressants.

This is the first prospective multicenter study concerning the real-life incidence of irAEs in solid tumor patients admitted in ICU. Despite the prospective design, identifying the imputability of ICIs in ICU admissions remains a challenge. To limit the bias, diagnosis was confirmed by both the intensivist and the oncologist at ICU admission and ICU discharge. Furthermore, every case was reviewed by two independent intensivists and one oncologist. Unfortunately, the diagnostic strategy could not be described in this study.

Several ICIs were studied, but anti-PD-(L)1s accounted for 95% of the patients. This ICI remained the most frequently used in cancer treatment during this period. Furthermore, association with other ICIs is used, particularly in melanoma, and will be increasingly prescribed in the coming years.

We identified an ICU admission “likely” associated with irAE in half of the patients at ICU admission and in 38% at ICU discharge. Intensivists easily suggest this diagnosis of irAE at ICU admission in patients treated with ICI. The knowledge of the whole situation during the ICU stay rectify the diagnosis in 20% of cases. Joseph et al. [4] reported the data of 112 cancer patients who received ICIs and were admitted in ICU within 60 days after the last dose. ICU admission was considered related to irAE in 26% of patients, other intercurrent event in 35%, or complications related to tumor progression in 39%. In a retrospective study conducted in 351 patients treated with an ICI [7], 129 (37%) had at least one presentation to the emergency department. This emergency department visit was related to irAE for 23 (18%) patients, infection for 25 (19%) patients, and progression/pain for 45 (35%) patients. Twelve patients were hospitalized and four required ICU care. In clinical trials, severe adverse events (CTCAE grade ≥ 3) were reported in 1 to 10% of patients [8].

Factors associated with ICU admission “likely” related to irAE were ECOG-PS and past history of irAE. This analysis was not performed in previous studies. Nevertheless, they are relevant when looking at the literature. irAE was described as potentially associated with better efficacy of ICIs (better ECOG-PS) [9, 10]. Furthermore, Kroschinski et al. [11] reported that in patients at risk for pneumonitis, the presence of further symptoms of irAE (skin, colitis, liver, or endocrine dysfunction) oriented the diagnosis. Moreover, in case of tumor progression, patients had a poorer ECOG-PS. Such patients with poor ECOG-PS at ICU admission were then at high risk of tumor progression.

IrAEs were mostly related to pneumonitis in this study (56%, n = 31/55), contrary to Joseph’ study [4]. Investigators in our study were both intensivists and pulmonologists. That explains the higher proportion of lung cancer and therefore of respiratory failure. In our cohort, cardiovascular failure was considered as an irAE in only 31% of cases at ICU admission and only 8% (n = 1/13) at ICU discharge. In multivariate analysis, it was preferentially associated with ICU admission “unlikely” related to irAE. Although immune myocarditis is a rare irAE, attributable mortality is very high, particularly in case of combination of anti-PD-(L)1 and anti-CTLA4 agents [12, 13]. Physician should assess the diagnosis quickly in order to introduce steroids as soon as possible [14].

In our cohort, 56% of patients with ICU admission “likely” related to irAE at ICU admission received steroids. Regarding imputability at ICU discharge, all patients with a certain or probable irAE received steroids. For three of them, they were started before ICU admission, and for 20 patients, on the day of ICU admission. Only three patients were treated with other immunosuppressants. Joseph et al. reported that 62% of patients admitted in ICU for irAE received steroids. In the study by Holstead et al. [7], all the patients required immunosuppressive therapy, and the median time to first dose of corticosteroid was 30.5 h (range 1–269). These results confirm that the use of steroids for irAE is a standard of care, as recommended [2, 3].

## Conclusions

In our cohort, irAEs accounted for half of ICU admissions in cancer patients receiving ICIs. Identifying the imputability of ICIs in ICU admissions remains a challenge. IrAEs were associated with a good ECOG-PS (0 or 1) and a past history of irAE. These patients with manifest or suspected irAE should be managed in strong collaboration between intensivists, oncologists, and organ specialists. Steroids were required in most cases.

## Supplementary Information

**Additional file 1: Table S1.** World Health Organization-Uppsala Monitoring Centre (WHO-UMC) causality categories. **Table S2.** Diagnosis at ICU admission according to proposed IrAE causality at ICU discharge (n = 110 patients).

## Data Availability

Data are available upon request from the corresponding author.

## Abbreviations

CTCAE: Common Terminology Criteria for Adverse Events
CTLA4: Cytotoxic T-lymphocyte antigen-4
ECOG: Eastern Cooperative Oncology Group
ICI: Immune checkpoint inhibitor
ICU: Intensive care unit
IQR: Interquartile range
irAE: Immune-related adverse event
PD-1: Programmed cell death 1
PD-L1: Programmed death-ligand 1
PS: Performance status
SAPS II: Simplified Acute Physiology Score II
WHO-UMC: World Health Organization-Uppsala Monitoring Centre
