# Supplementary material for: ICU admission for solid cancer patients treated with immune checkpoint inhibitors
Source: Ann Intensive Care. 2023 Apr 18;13:29. doi: 10.1186/s13613-023-01122-z (PMC10113402; doi:10.1186/s13613-023-01122-z)
Supplement: Supplementary file 1 — Additional file 1: Table S1. World Health Organization-Uppsala Monitoring Centre (WHO-UMC) causality categories. Table S2. Diagnosis at ICU admission according to proposed IrAE causality at ICU discharge (n = 110 patients). [file 13613_2023_1122_MOESM1_ESM.docx]

**Additional file**

**Table S1** World Health Organization-Uppsala Monitoring Centre (WHO-UMC) causality categories

| Causality term | Assessment criteria* |
| --- | --- |
| Certain | • Event or laboratory test abnormality, with plausible time relationship to drug intake • Cannot be explained by disease or other drugs • Response to withdrawal plausible (pharmacologically, pathologically) • Event definitive pharmacologically or phenomenologically (i.e. an objective and specific medical disorder or a recognized pharmacological phenomenon) • Rechallenge satisfactory, if necessary |
| Probable / Likely | • Event or laboratory test abnormality, with reasonable time relationship to drug intake • Unlikely to be attributed to disease or other drugs • Response to withdrawal clinically reasonable • Rechallenge not required |
| Possible | • Event or laboratory test abnormality, with reasonable time relationship to drug intake • Could also be explained by disease or other drugs • Information on drug withdrawal may be lacking or unclear |
| Unlikely | • Event or laboratory test abnormality, with a time to drug intake that makes a relationship improbable (but not impossible) • Disease or other drugs provide plausible explanations |
| Conditional / Unclassified | • Event or laboratory test abnormality • More data for proper assessment needed, or • Additional data under examination |
| Unassessable / Unclassifiable | • Report suggesting an adverse reaction • Cannot be judged because information is insufficient or contradictory • Data cannot be supplemented or verified |

* All points should be reasonably complied with

**Table S2** Diagnosis at ICU admission according to proposed IrAE causality at ICU discharge (n=110 patients)

|  | All n=110 | Unlikely causality n=66 (60) | Likely causality n=44 (38) |
| --- | --- | --- | --- |
| Respiratory | 66 (60) | 38 (58) | 28 (64) |
| - pneumopathy | 52 | 27 | 25 |
| - tumor obstruction/invasion (pleural effusion, hemorrhage, superior vena cava syndrome) | 11 | 9 | 2 |
| - bronchospasm | 3 | 2 | 1 |
| Colitis | 14 (13) | 8 (12) | 6 (14) |
| Cardiovascular | 13 (12) | 12 (18) | 1 (2) |
| - pericardial effusion | 3 | 3 | 0 |
| - rhythm disorder | 2 | 2 | 0 |
| - myocarditis | 1 | 0 | 1 |
| - other (hypotension, pulmonary embolism) | 7 | 7 | 0 |
| Metabolic | 11 (10) | 5 (8) | 6 (14) |
| - renal failure | 6 | 4 | 2 |
| - corticotropic insufficiency | 2 | 0 | 2 |
| - hyponatremia | 1 | 0 | 1 |
| - hepatitis | 1 | 1 | 0 |
| - thrombotic microangiopathy | 1 | 0 | 1 |
| Neurological | 6 (5) | 3 (5) | 3 (7) |
| - coma | 2 | 2 | 0 |
| - encephalitis | 2 | 1 | 1 |
| - myositis | 1 | 0 | 1 |
| - myasthenia | 1 | 0 | 1 |

Qualitative variables are expressed as n (%) and quantitative variables as median [interquartile range 25%–75%].

*ICU* intensive care unit, *Miss*. missing data, *SAPS II* Simplified Acute Physiology Score II.
